# Optimizing taxonomic classification of marker-gene amplicon sequences with QIIME 2’s q2-feature-classifier plugin

**DOI:** 10.1186/s40168-018-0470-z

**Published:** 2018-05-17

**Authors:** Nicholas A. Bokulich, Benjamin D. Kaehler, Jai Ram Rideout, Matthew Dillon, Evan Bolyen, Rob Knight, Gavin A. Huttley, J. Gregory Caporaso

**Affiliations:** 1 The Pathogen and Microbiome Institute, Northern Arizona University, PO Box 4073, Flagstaff, AZ 86011-4073 USA; 2 Research School of Biology, Australian National University, 46 Sullivans Creek Road, Acton ACT, 2601 Australia; 3 Departments of Pediatrics and Computer Science and Engineering, and Center for Microbiome Innovation, University of California San Diego, La Jolla, CA USA; 4 Department of Biological Sciences, Northern Arizona University, 1298 S Knoles Drive, Building 56, 3rd Floor, Flagstaff, AZ USA

## Abstract

**Background:**

Taxonomic classification of marker-gene sequences is an important step in microbiome analysis.

**Results:**

We present q2-feature-classifier (https://github.com/qiime2/q2-feature-classifier), a QIIME 2 plugin containing several novel machine-learning and alignment-based methods for taxonomy classification. We evaluated and optimized several commonly used classification methods implemented in QIIME 1 (RDP, BLAST, UCLUST, and SortMeRNA) and several new methods implemented in QIIME 2 (a scikit-learn naive Bayes machine-learning classifier, and alignment-based taxonomy consensus methods based on VSEARCH, and BLAST+) for classification of bacterial 16S rRNA and fungal ITS marker-gene amplicon sequence data. The naive-Bayes, BLAST+-based, and VSEARCH-based classifiers implemented in QIIME 2 meet or exceed the species-level accuracy of other commonly used methods designed for classification of marker gene sequences that were evaluated in this work. These evaluations, based on 19 mock communities and error-free sequence simulations, including classification of simulated “novel” marker-gene sequences, are available in our extensible benchmarking framework, tax-credit (https://github.com/caporaso-lab/tax-credit-data).

**Conclusions:**

Our results illustrate the importance of parameter tuning for optimizing classifier performance, and we make recommendations regarding parameter choices for these classifiers under a range of standard operating conditions. q2-feature-classifier and tax-credit are both free, open-source, BSD-licensed packages available on GitHub.

## Background

High-throughput sequencing technologies have transformed our ability to explore complex microbial communities, offering insight into microbial impacts on human health [1] and global ecosystems [2]. This is achieved most commonly by sequencing short, conserved marker genes amplified with ‘universal’ PCR primers, such as 16S rRNA genes for bacteria and archaea, or internal transcribed spacer (ITS) regions for fungi. Targeted marker-gene primers can also be used to profile specific taxa or functional groups, such as nifH genes [3]. These sequences often are compared against an annotated reference sequence database to determine the likely taxonomic origin of each sequence with as much specificity as possible. Accurate and specific taxonomic information is a crucial component of many experimental designs.

Challenges in this process include the short length of typical sequencing reads with current technology, sequencing and PCR errors [4], selection of appropriate marker genes that contain sufficient heterogeneity to differentiate target species but that are homogeneous enough in some regions to design broad-spectrum primers, quality of reference sequence annotations [5], and selection of a method that accurately predicts the taxonomic affiliation of millions of sequences at low computational cost. Numerous methods have been developed for taxonomy classification of DNA sequences, but few have been directly compared in the specific case of short marker-gene sequences.

We introduce q2-feature-classifier, a QIIME 2 (https://qiime2.org) plugin for taxonomy classification of marker-gene sequences. QIIME 2 is the successor to the QIIME [6] microbiome analysis package. The q2-feature-classifier plugin supports use of any of the numerous machine-learning classifiers available in scikit-learn [7, 8] for marker gene taxonomy classification, and currently provides two alignment-based taxonomy consensus classifiers based on BLAST+ [9] and VSEARCH [10]. We evaluate the latter two methods and the scikit-learn multinomial naive Bayes classifier (labeled “naive Bayes” in the “Results” section) for the first time. We show that the QIIME 2 classifiers provided in q2-feature-classifier match or outperform the classification accuracy of the widely used QIIME 1 methods for sequence classification, and that performance of the naive Bayes classifier can be significantly increased by providing it with information regarding expected taxonomic composition. Some of the taxonomy classification methods in QIIME 1 (RDP classifier [11] and BLAST [9]) are thin wrappers around the original software; other methods based on uclust [12] SortMeRNA [13](QIIME 1), VSEARCH, and BLAST+ (QIIME 2) are also wrapped implementations of other software followed by consensus taxonomic assignment by QIIME software. Thus, while our analyses focus on methods currently implemented in these versions of QIIME, we expect that the results will generalize to similar applications of those tools outside of QIIME.

We also developed tax-credit (https://github.com/caporaso-lab/tax-credit-code/ and https://github.com/caporaso-lab/tax-credit-data/), an extensible computational framework for evaluating taxonomy classification accuracy. This framework streamlines the process of methods benchmarking by compiling multiple different test data sets, including mock communities [14] and simulated sequence reads. It additionally stores pre-computed results from previously evaluated methods, including the results presented here, and provides a framework for parameter sweeps and method optimization. Tax-credit could be used as an evaluation framework by other research groups in the future or its raw data could be easily extracted for integration in another evaluation framework.

## Results

We used tax-credit to optimize and compare multiple marker-gene sequence taxonomy classifiers. We evaluated two commonly used classifiers that are wrapped in QIIME 1 (RDP Classifier (version 2.2) [11], legacy BLAST (version 2.2.22) [15]), two QIIME 1 alignment-based consensus taxonomy classifiers (the default UCLUST classifier available in QIIME 1 (based on version 1.2.22q) [12], and SortMeRNA (version 2.0 29/11/2014) [13]), two alignment-based consensus taxonomy classifiers newly released in q2-feature-classifier (based on BLAST+ (version 2.6.0) [9] and VSEARCH (version 2.0.3) [10]), and a new multinomial naive Bayes machine-learning classifier in q2-feature-classifier (see the “Methods” section for information about q2-feature-classifier methods and source code availability). We performed parameter sweeps to determine optimal parameter configurations for each method.

### Mock community evaluations

We first benchmarked classifier performance on mock communities, which are artificially constructed mixtures of microbial cells or DNA combined at known ratios [14]. We utilized 15 bacterial 16S rRNA gene mock communities and 4 fungal internal transcribed spacer (ITS) mock communities (Table 1) sourced from mockrobiota [14], a public repository for mock community data. Mock communities are useful for method benchmarking because (1) unlike for simulated communities, they allow quantitative assessments of method performance under actual operating conditions, i.e., incorporating real sequencing errors that can be difficult to model accurately; and (2) unlike for natural community samples, the actual composition of a mock community is known in advance, allowing quantitative assessments of community profiling accuracy.

**Table 1:** Mock communities currently integrated in tax-credit

Study ID^a^	Target gene^b^	Platform	Species	Strains	Citation
mock-1	16S	HiSeq	46	48	[33]
mock-2	16S	MiSeq	46	48	[33]
mock-3	16S	MiSeq	21	21	[33]
mock-4	16S	MiSeq	21	21	[33]
mock-5	16S	MiSeq	21	21	[33]
mock-7	16S	HiSeq	67	67	[34]
mock-8	16S	HiSeq	67	67	[14]
mock-9	ITS	HiSeq	13	16	[14]
mock-10	ITS	HiSeq	13	16	[14]
mock-12	16S	MiSeq	26	27	[4]
mock-16	16S	MiSeq	56	59	[35]
mock-18	16S	MiSeq	15	15	[36]
mock-19	16S	MiSeq	15	27	[36]
mock-20	16S	MiSeq	20	20	[37]
mock-21	16S	MiSeq	20	20	[37]
mock-22	16S	MiSeq	20	20	[37]
mock-23	16S	MiSeq	20	20	[37]
mock-24	ITS	MiSeq	8	8	[38]
mock-26	ITS	FLX Titanium	11	11	[39]

^a^All studies are available on mockrobiota [14] at https://github.com/caporaso-lab/mockrobiota/tree/master/data/[studyID]

^b^Abbreviations: *16S,* 16S rRNA gene; *HiSeq*, Illumina HiSeq; *MiSeq*, Illumina MiSeq

An additional priority was to test the effect of setting class weights on classification accuracy for the naive Bayes classifier implemented in q2-feature-classifier. In machine learning, class weights or prior probabilities are vectors of weights that specify the frequency at which each class is expected to be observed (and should be distinguished from the use of this term under Bayesian inference as a probability distribution of weights vectors). An alternative to setting class weights is to assume that each query sequence is equally likely to belong to any of the taxa that are present in the reference sequence database. This assumption, known as uniform class priors in the context of a naive Bayes classifier, is made by the RDP classifier [11], and its impact on marker-gene classification accuracy has yet to be validated. Making either assumption that the class weights are uniform or known to some extent will affect results and cannot be avoided. The mock communities have taxonomic abundances that are far from uniform over the set of reference taxonomies, as any real data set must. We can therefore use them to assess the impact of making assumptions regarding class weights. Where we have set the class weights to the known taxonomic composition of a sample, we have labeled the results “bespoke”.

**Fig. 1:**
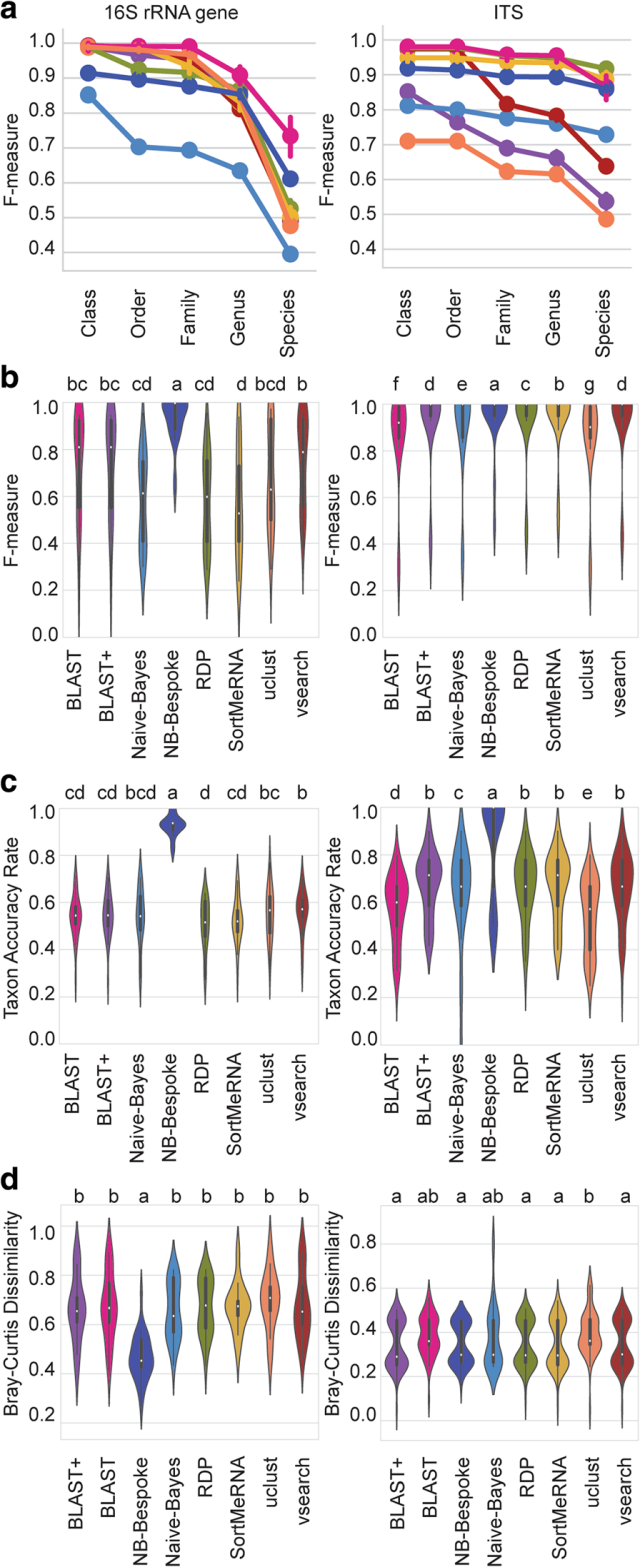
Classifier performance on mock community datasets for 16S rRNA gene sequences (left column) and fungal ITS sequences (right column). **a** Average F-measure for each taxonomy classification method (averaged across all configurations and all mock community datasets) from class to species level. Error bars = 95% confidence intervals. **b** Average F-measure for each optimized classifier (averaged across all mock communities) at species level. **c** Average taxon accuracy rate for each optimized classifier (averaged across all mock communities) at species level. **d** Average Bray-Curtis distance between the expected mock community composition and its composition as predicted by each optimized classifier (averaged across all mock communities) at species level. Violin plots show median (white point), quartiles (black bars), and kernel density estimation (violin) for each score distribution. Violins with different lower-case letters have significantly different means (paired *t* test false detection rate-corrected *P* < 0.05)

We evaluated classifier performance accuracy on mock community sequences classified at taxonomic levels from class through species. Mock community sequences were classified using the Greengenes 99% OTUs 16S rRNA gene or UNITE 99% OTUs ITS reference sequences for bacterial and fungal mock communities, respectively. As expected, classification accuracy decreased as classification depth increased, and all methods could predict the taxonomic affiliation of mock community sequences down to genus level with median F-measures exceeding 0.8 across all parameter sets (minimum: UCLUST *F* = 0.81, maximum: naive Bayes bespoke *F* = 1.00) (Fig. 1a). However, species affiliation was predicted with much lower and more variable accuracy among method configurations (median F-measure minimum: UCLUST F = 0.42, maximum: naive Bayes bespoke *F* = 0.95), highlighting the importance of parameter optimization (discussed in more detail below). Figure 1a illustrates line plots of mean F-measure at each taxonomic level, averaged across all classifier configurations; hence, classifier performance is underestimated for some classifiers that are strongly affected by parameter configurations or for which a wider range of parameters were tested (e.g., naive Bayes). Comparing only optimized methods (i.e., the top-performing parameter configurations for each method), naive Bayes bespoke achieved significantly higher F-measure (paired *t* test *P* < 0.05) (Fig. 1b), recall, taxon detection rate, taxon accuracy rate (Fig. 1c), and lower Bray-Curtis dissimilarity than all other methods (Fig. 1d).

Mock communities are necessarily simplistic, and cannot assess method performance across a diverse range of taxa. Although raw sequences may contain PCR and sequencing errors (allowing us to assess method performance under biological conditions), sequences that do match the expected mock community sequences are not removed from the reference database prior to classification. This approach replicates normal operating conditions and assesses recovery of expected sequences, but may implicitly bias toward methods that find an exact match to the query sequences, and does not approximate some natural microbial communities in which few or no detected sequences exactly match the reference sequences. Hence, we performed simulated sequence read classifications (described below) to further test classifier performance.

### Cross-validated taxonomy classification

Simulated sequence reads, derived from reference databases, allow us to assess method performance across a greater diversity of sequences than a single mock community generally encompasses. We first evaluated classifier performance using stratified k-fold cross-validation of taxonomy classification for simulated reads. The k-fold cross-validation strategy is modified slightly to account for the hierarchical nature of taxonomic classifications, which all of the classifiers in this study (with the exception of legacy BLAST) handle by assigning the lowest (i.e., most specific) taxonomic level where the classification surpasses some user-defined “confidence” or “consensus” threshold (see materials and methods). The modification is to truncate any expected taxonomy in each test set to the maximum level at which an instance of that taxonomy exists in the training set.

Simulated reads were generated from Greengenes 99% OTUs 16S rRNA gene or UNITE 99% OTUs ITS reference sequences. Greengenes 16S rRNA gene simulated reads were generated from full-length 16S rRNA genes (primers 27F/1492R) and V4 (primers 515F/806R) and V1–3 subdomains (primers 27F/534R). The simulated reads currently available in tax-credit do not incorporate artificial errors from PCR or sequencing for several reasons. As our mock communities analyses already assess classifier performance under true noisy experimental conditions, the goal of the analyses of simulated sequences is to assess theoretical classifier performance (when exact sequence matches do not exist in the reference database). Additionally, marker-gene amplicon sequence analysis pipelines commonly utilize denoising methods [4] to model per-run error profiles, filter noisy sequences, and resolve actual sequence variants. Hence, in our evaluations, we simulate an idealized (if unlikely) theoretical scenario in which all sequencing errors have been denoised in order to separate classifier performance from denoiser performance. In this set of tests and below for novel taxa, the “bespoke” classifier had prior probabilities that were inferred from the training set each time it was trained.

**Fig. 2:**
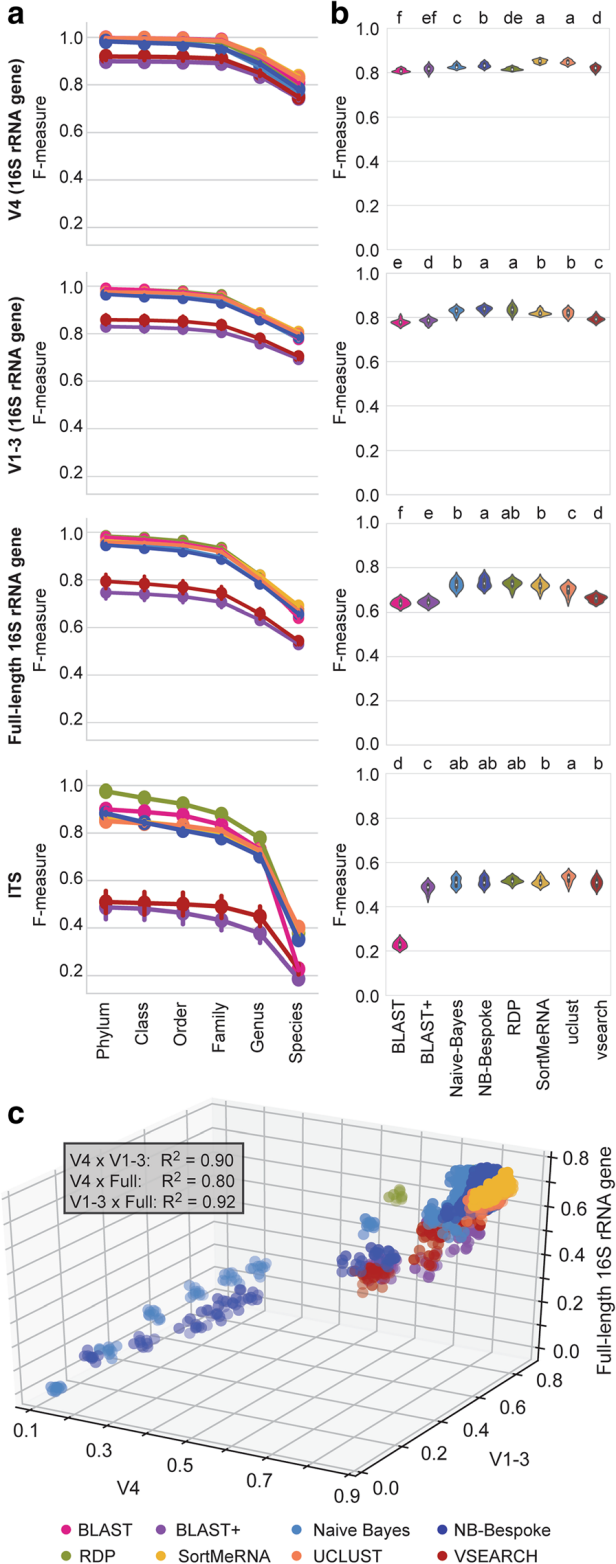
Classifier performance on cross-validated sequence datasets. Classification accuracy of 16S rRNA gene V4 subdomain (first row), V1–3 subdomain (second row), full-length 16S rRNA gene (third tow), and fungal ITS sequences (fourth row). **a** Average F-measure for each taxonomy classification method (averaged across all configurations and all cross-validated sequence datasets) from class to species level. Error bars = 95% confidence intervals. **b** Average F-measure for each optimized classifier (averaged across all cross-validated sequence datasets) at species level. Violins with different lower-case letters have significantly different means (paired t-test false detection rate-corrected *P* < 0.05). **c** correlation between F-measure performance for each method/configuration classification of V4 subdomain (*x* axis), V1–3 subdomain (*y* axis), and full-length 16S rRNA gene sequences (*z* axis). Inset lists the Pearson *R*^2^ value for each pairwise correlation; each correlation is significant (*P* < 0.001)

Classification of cross-validated reads performed better at coarser levels of classification (Fig. 2a), similar to the trend observed in mock community results. For bacterial sequences, average classification accuracy for all methods declined from near-perfect scores at family level (V4 domain median F-measure minimum: BLAST+ *F* = 0.92, maximum: legacy BLAST *F* = 0.99), but still retained accurate scores at species level (median minimum: BLAST+ *F* = 0.76, maximum: SortMeRNA *F* = 0.84), relative to some mock community data sets (Fig. 2a). Fungal sequences exhibited similar performance, with the exception that mean BLAST+ and VSEARCH performance was markedly lower at all taxonomic levels, indicating high sensitivity to parameter configurations, and species-level F-measures were in general much lower (median minimum: BLAST+ *F* = 0.17, maximum: UCLUST *F* = 0.45) than those of bacterial sequence classifications (Fig. 2a).

Species-level classifications of 16S rRNA gene simulated sequences were best with optimized UCLUST and SortMeRNA configurations for V4 domain, and naive Bayes and RDP for V1–3 domain and full-length 16S rRNA gene sequences (Fig. 2b). UCLUST achieved the highest F-measure for ITS classification (*F* = 0.51). However, all optimized classifiers achieved similar F-measure ranges, with the exception of legacy BLAST for ITS sequences (Fig. 2b).

Species-level classification performance of 16S rRNA gene simulated reads was significantly correlated between each subdomain and the full-length gene sequences (Fig. 2c). In our tests, full-length sequences exhibited slightly lower accuracy than V1–3 and V4 subdomains. The relative performance of full-length 16S rRNA genes versus hypervariable subdomain reads is variable in the literature [11, 16–21], and our results add another data point to the ongoing discussion of this topic. Nevertheless, species-level classifications yielded strong correlation between method configurations (Fig. 2c) and optimized method performance (Fig. 2b), suggesting that primer choice impacts classification accuracy uniformly across all methods. Hence, we focused on V4 subdomain reads for downstream analyses.

### Novel taxon classification evaluation

Novel taxon classification offers a unique perspective on classifier behavior, assessing how classifiers perform when challenged with a “novel” clade that is not represented in the reference database [22–25]. An ideal classifier should identify the nearest taxonomic lineage to which this taxon belongs, but no further. In this evaluation, a reference database is subsampled k times to generate query and reference sequence sets, as for cross-validated classification, but two important distinctions exist: (1) the reference database used for classification excludes any sequence that matches the taxonomic affiliation of the query sequences at taxonomic level *L*, the taxonomic rank at which classification is being attempted; and (2) this is performed at each taxonomic level, in order to assess classification performance when each method encounters a “novel” species, genus, family, etc.

Due to these differences, interpretation of novel taxon classification results is different from that of mock community and cross-validated classifications. For the latter, classification accuracy may be assessed at each taxonomic level for each classification result: mean classification accuracy at family level and species level evaluate the same results but focus on different taxonomic levels of classification. For novel taxa, however, different query and reference sequences are compiled for classification at each taxonomic level and separate classifications are performed for each. Hence, classifications at family and species level are independent events—one assesses how accurately each method performs when it encounters a “novel” family that is not represented in the reference database, the other when a “novel” species is encountered.

Novel taxon evaluations employ a suite of modified metrics to provide more information on what types of classification errors occur. Precision, recall, and F-measure calculations at each taxonomic level *L* assess whether an accurate taxonomy classification was made at level *L*-1: for example, a “novel” species should be assigned a genus, because the correct species class is not represented within the reference database. Any species-level classification in this scenario is an *overclassification* (affecting both recall and precision) [25]. Overclassification is one of the key metrics for novel taxa evaluation, indicating the degree to which novel sequences will be misinterpreted as known organisms. This overclassification is often highly undesirable because it can lead, for example, to the incorrect classification of unknown but most likely innocuous environmental sequences as known pathogens. Novel sequences that are classified within the correct clade, but to a less specific level than *L,* are *underclassified* (affecting recall but not precision) [25]. Sequences that are classified into a completely different clade are *misclassified* (affecting both recall and precision) [25].

**Fig. 3:**
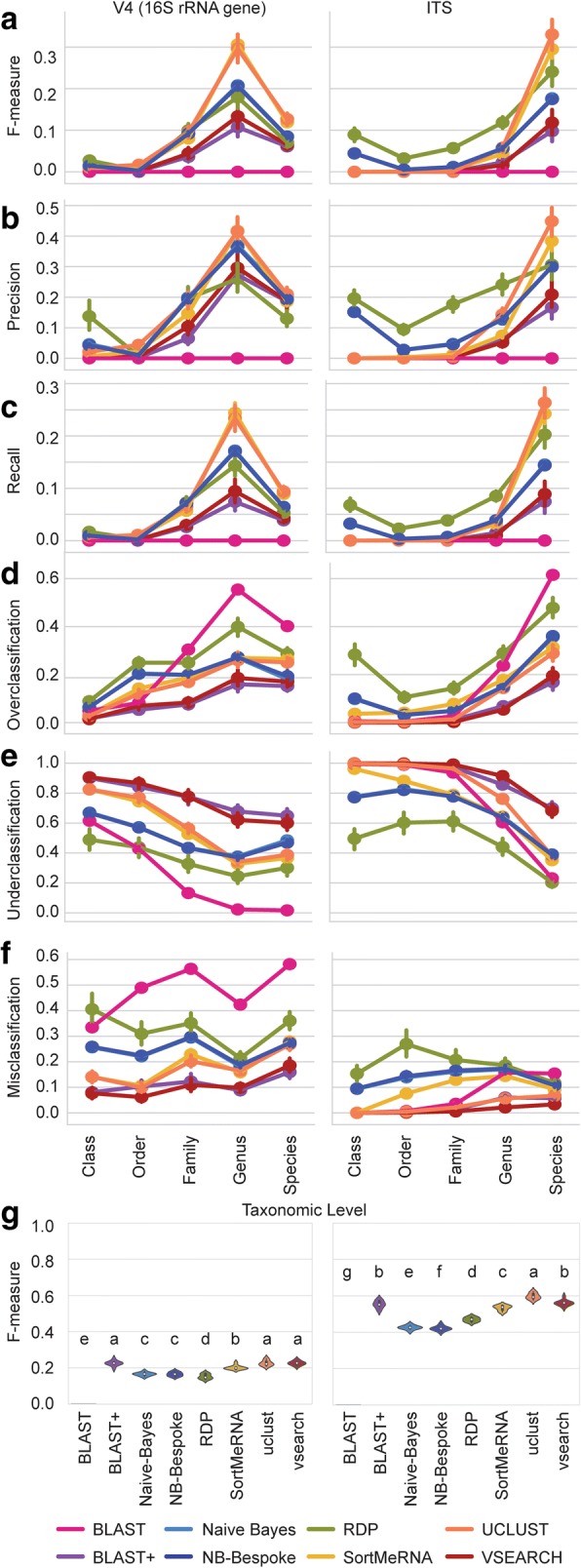
Classifier performance on novel-taxa simulated sequence datasets for 16S rRNA gene sequences (left column) and fungal ITS sequences (right column). **a**–**f**, Average F-measure (**a**), precision (**b**), recall (**c**), overclassification (**d**), underclassification (**e**), and misclassification (**f**) for each taxonomy classification method (averaged across all configurations and all novel taxa sequence datasets) from phylum to species level. Error bars = 95% confidence intervals. **b** Average F-measure for each optimized classifier (averaged across all novel taxa sequence datasets) at species level. Violins with different lower-case letters have significantly different means (paired *t* test false detection rate-corrected *P* < 0.05)

Precision, recall, and F-measure all gradually increase from average scores near 0.0 at class level, reaching peak scores at genus level for bacteria and species level for fungi (Fig. 3a–c). These trends are paired with gradual decreases in underclassification and misclassification rates for all classification methods, indicating that all classifiers perform poorly when they encounter sequences with no known match at the class, order, or family levels (Fig. 3d, f). At species level, UCLUST, BLAST+, and VSEARCH achieved significantly better F-measures than all other methods for 16S rRNA gene classifications (*P* < 0.05) (Fig. 3g). UCLUST achieved significantly better F-measures than all other methods for ITS classifications (Fig. 3g). Over-, under-, and misclassification scores are less informative for optimizing classifiers for real use cases, as most methods could be optimized to yield near-zero scores for each of these metrics separately, but only through extreme configurations, leading to F-measures that would be unacceptable under any scenario. Note that all comparisons were made between methods optimized to maximize (or minimize) a single metric, and hence the configurations that maximize precision are frequently different from those that maximize recall or other metrics. This trade-off between different metrics is discussed in more detail below.

The novel taxon evaluation provides an estimate of classifier performance given a specific reference database, but its generalization is limited by the quality of the reference databases available and by the label-based approach used for partitioning and evaluation. Mislabeled and polyphyletic clades in the database, e.g., clostridium group, increase the probability of misclassification. A complementary analysis based on sequence similarity between a novel query and top reference hit could mitigate this issue. However, we choose to apply a label-based approach, as it better reflects the biological problem that users can expect to encounter, i.e., using a particular reference sequence database (which will contain some quantity of mislabeled and polyphyletic taxa inherent to currently available resources), how likely is a classifier to misclassify a taxonomic label?

### Multi-evaluation method optimization

**Fig. 4:**
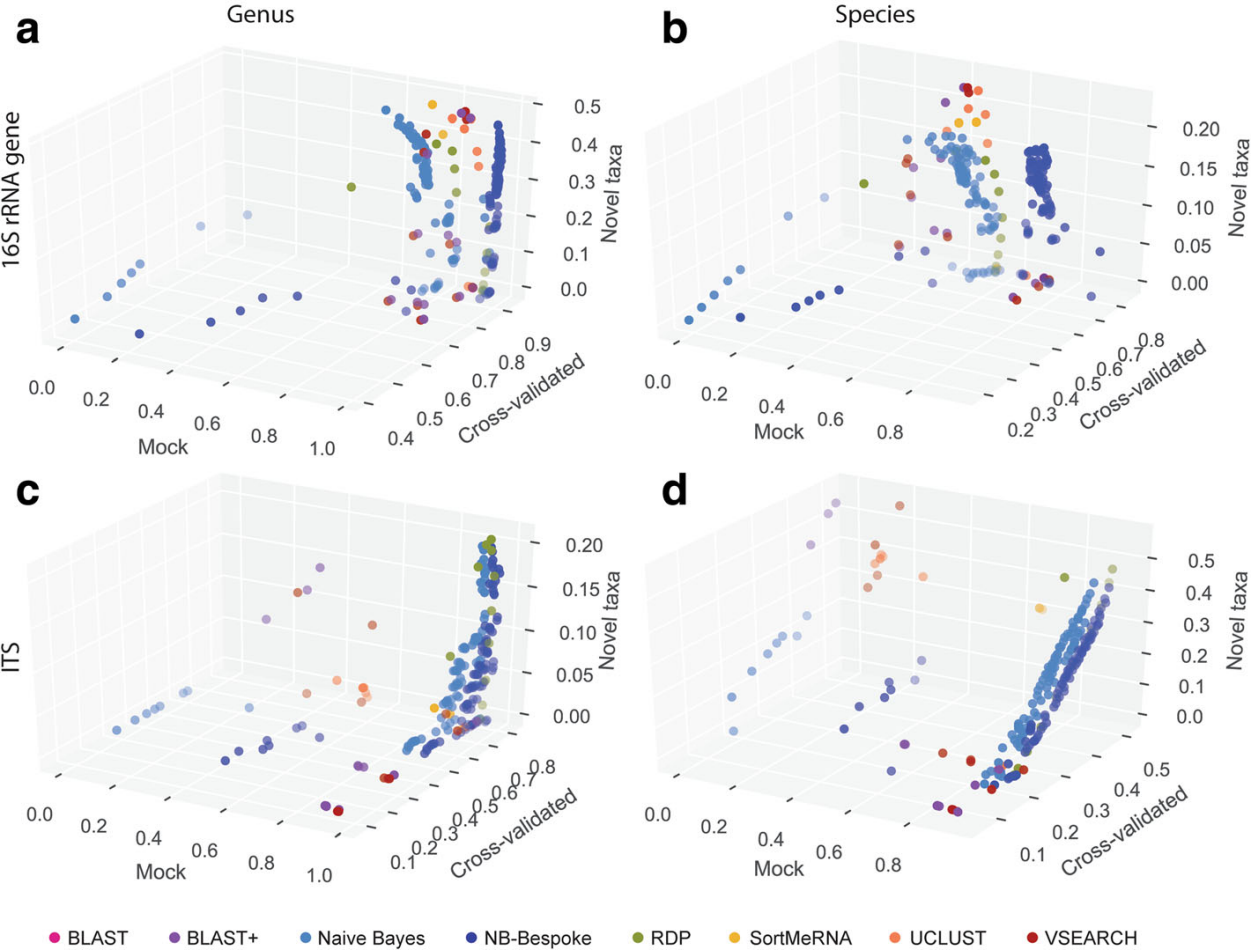
Classification accuracy comparison between mock community, cross-validated, and novel taxa evaluations. Scatterplots show mean F-measure scores for each method configuration, averaged across all samples, for classification of 16S rRNA genes at genus level (**a**) and species level (**b**), and fungal ITS sequences at genus level (**c**) and species level (**d**)

The mock community and cross-validation classification evaluations yielded similar trends in configuration performance, but optimizing parameters choices for the novel taxa generally led to suboptimal choices for the mock community and cross-validation tests (Fig. 4). We sought to determine the relationship between method configuration performance for each evaluation and use this information to select configurations that perform best across all evaluations. For 16S rRNA gene sequence species-level classification, method configurations that achieve maximum F-measures for mock and cross-validated sequences can perform poorly for novel taxon classification (Fig. 4b). Optimization is more straightforward for genus-level classification of 16S rRNA gene sequences (Fig. 4a) and for fungal sequences (Fig. 4c, d), for which configuration performance (measured as mean F-measure) is maximized by similar configurations among all three evaluations.

To identify optimal method configurations, we set accuracy score minimum thresholds for each evaluation by identifying natural breaks in the range of quality scores, selecting methods and parameter ranges that met these criteria. Table 2 lists method configurations that maximize species-level classification accuracy scores for mock community, cross-validated, and novel taxon evaluations under several common operating conditions. “Balanced” configurations are recommended for general use and are methods that maximize F-measure scores. “Precision” and “recall” configurations maximize precision and recall scores, respectively, for mock, cross-validated, and novel-taxa classifications (Table 2). “Novel” configurations optimize F-measure scores for novel taxon classification, and secondarily for mock and cross-validated performance (Table 2). These configurations are recommended for use with sample types that are expected to contain large proportions of unidentified species, for which overclassification can be excessive. However, these configurations may not perform optimally for classification of known species (i.e., underclassification rates will be higher). For fungi, the same configurations recommended for “precision” perform well for novel taxon classification (Table 2). For 16S rRNA gene sequences, BLAST+, UCLUST, and VSEARCH consensus classifiers perform best for novel taxon classification (Table 2).

**Table 2:** Optimized methods configurations for standard operating conditions

				Mock			Cross-validated			Novel taxa			
Target	Condition	Method	Parameters	*F*	*P*	*R*	*F*	*P*	*R*	*F*	*P*	*R*	Threshold
16S rRNA gene	Balanced	NB-bespoke	[6,6]:0.9	0.705	0.98	0.582	0.827	0.931	0.744	0.165	0.243	0.125	*F* = (0.49, 0.8, 0.1)
			[6,6]:0.92	0.705	0.98	0.581	0.825	0.936	0.737	0.165	0.251	0.123	*F* = (0.7, 0.8, 0.15)
			[6,6]:0.94	0.703	0.98	0.579	0.822	0.942	0.729	0.162	0.259	0.118	
			[7,7]:0.92	0.712	0.978	0.592	0.831	0.931	0.751	0.151	0.221	0.115	
			[7,7]:0.94	0.708	0.978	0.586	0.829	0.936	0.743	0.157	0.239	0.117	
		Naive-Bayes	[7,7]:0.7	0.495	0.797	0.38	0.819	0.886	0.761	0.115	0.138	0.099	
		rdp	0.6	0.564	0.798	0.457	0.815	0.868	0.768	0.102	0.128	0.084	
			0.7	0.55	0.799	0.438	0.812	0.892	0.746	0.124	0.173	0.096	
		Uclust	0.51:0.9:3	0.498	0.746	0.392	0.846	0.876	0.817	0.154	0.201	0.126	
	Precision	NB-bespoke	[6,6]:0.98	0.676	0.987	0.537	0.803	0.956	0.692	0.163	0.303	0.111	*P* = (0.94, 0.95, 0.25)
			[7,7]:0.98	0.687	0.98	0.551	0.815	0.951	0.713	0.164	0.283	0.115	
		rdp	1	0.239	0.941	0.16	0.632	0.968	0.469	0.12	0.457	0.069	
	Recall	NB-bespoke	[12,12]:0.5	0.754	0.8	0.721	0.815	0.83	0.801	0.053	0.058	0.049	*R* = (0.47, 0.75, 0.04)
			[14,14]:0.5	0.758	0.802	0.726	0.811	0.826	0.797	0.052	0.057	0.048	*R* = (0.7, 0.75, 0.04)
			[16,16]:0.5	0.755	0.785	0.732	0.808	0.825	0.792	0.052	0.058	0.047	
			[18,18]:0.5	0.772	0.803	0.748	0.805	0.823	0.789	0.055	0.061	0.05	
			[32,32]:0.5	0.937	0.966	0.913	0.788	0.818	0.76	0.054	0.067	0.045	
		Naive-Bayes	[11,11]:0.5	0.567	0.77	0.479	0.793	0.82	0.768	0.059	0.065	0.055	
			[12,12]:0.5	0.567	0.769	0.479	0.79	0.816	0.765	0.059	0.064	0.055	
			[18,18]:0.5	0.564	0.764	0.477	0.779	0.807	0.753	0.057	0.063	0.051	
		rdp	0.5	0.577	0.791	0.48	0.816	0.848	0.787	0.068	0.079	0.06	
	Novel	Blast+	10:0.51:0.8	0.436	0.723	0.325	0.816	0.896	0.749	0.225	0.332	0.171	*F* = (0.4, 0.8, 0.2)
		Uclust	0.76:0.9:5	0.467	0.775	0.348	0.84	0.938	0.76	0.219	0.358	0.158	
		VSEARCH	10:0.51:0.8	0.45	0.74	0.342	0.814	0.891	0.75	0.226	0.333	0.171	
			10:0.51:0.9	0.45	0.74	0.342	0.82	0.896	0.755	0.219	0.338	0.162	
Fungi	Balanced	Naive-Bayes	[6,6]:0.94	0.874	0.935	0.827	0.481	0.57	0.416	0.374	0.438	0.327	*F* = (0.85, 0.45, 0.37)
			[6,6]:0.96	0.874	0.935	0.827	0.495	0.597	0.423	0.399	0.473	0.344	
			[6,6]:0.98	0.874	0.935	0.827	0.505	0.629	0.423	0.426	0.52	0.361	
			[7,7]:0.98	0.874	0.935	0.827	0.485	0.596	0.409	0.388	0.47	0.33	
		NB-bespoke	[6,6]:0.94	0.928	0.968	0.915	0.48	0.567	0.416	0.371	0.433	0.325	
			[6,6]:0.96	0.928	0.968	0.915	0.491	0.59	0.42	0.393	0.466	0.34	
			[6,6]:0.98	0.927	0.97	0.913	0.504	0.624	0.422	0.421	0.512	0.358	
			[7,7]:0.98	0.935	0.97	0.921	0.487	0.596	0.412	0.386	0.466	0.329	
		rdp	0.7	0.929	0.939	0.922	0.479	0.572	0.413	0.382	0.451	0.332	
			0.8	0.924	0.939	0.915	0.507	0.633	0.422	0.434	0.534	0.366	
			0.9	0.922	0.937	0.913	0.517	0.698	0.411	0.47	0.617	0.379	
	Precision	Naive-Bayes	[6,6]:0.98	0.874	0.935	0.827	0.505	0.629	0.423	0.426	0.52	0.361	*P* = (0.92, 0.6, 0.3)
		NB-bespoke	[6,6]:0.98	0.927	0.97	0.913	0.504	0.624	0.422	0.421	0.512	0.358	
		rdp	0.8	0.924	0.939	0.915	0.507	0.633	0.422	0.434	0.534	0.366	
			0.9	0.922	0.937	0.913	0.517	0.698	0.411	0.47	0.617	0.379	
			1	0.821	0.943	0.742	0.461	0.81	0.322	0.459	0.774	0.327	
	Recall	NB-bespoke	[6,6]:0.92	0.938	0.971	0.924	0.467	0.544	0.409	0.353	0.407	0.312	*R* = (0.9, 0.4, 0.3)
			[6,6]:0.94	0.928	0.968	0.915	0.48	0.567	0.416	0.371	0.433	0.325	
			[6,6]:0.96	0.928	0.968	0.915	0.491	0.59	0.42	0.393	0.466	0.34	
			[6,6]:0.98	0.927	0.97	0.913	0.504	0.624	0.422	0.421	0.512	0.358	
			[7,7]:0.96	0.935	0.969	0.921	0.47	0.56	0.404	0.357	0.422	0.31	
			[7,7]:0.98	0.935	0.97	0.921	0.487	0.596	0.412	0.386	0.466	0.329	
		rdp	0.7	0.929	0.939	0.922	0.479	0.572	0.413	0.382	0.451	0.332	
			0.8	0.924	0.939	0.915	0.507	0.633	0.422	0.434	0.534	0.366	
			0.9	0.922	0.937	0.913	0.517	0.698	0.411	0.47	0.617	0.379	
	Novel	Naive-Bayes	[6,6]:0.98	0.874	0.935	0.827	0.505	0.629	0.423	0.426	0.52	0.361	*F* = (0.85, 0.45, 0.4)
		NB-bespoke	[6,6]:0.98	0.927	0.97	0.913	0.504	0.624	0.422	0.421	0.512	0.358	
		rdp	0.8	0.923	0.939	0.915	0.507	0.633	0.422	0.434	0.534	0.366	
			0.9	0.921	0.937	0.913	0.517	0.698	0.411	0.47	0.617	0.379	

^a^*F*, F-measure; *P,* precision; *R*, recall

^b^Naive Bayes parameters: k-mer range, confidence

^c^RDP parameters: confidence

^d^BLAST+/VSEARCH parameters: max accepts, minimum consensus, minimum percent identity

^e^UCLUST parameters: minimum consensus, similarity, max accepts

^f^Threshold describes the score cut-offs used to define optimal method ranges, in the following format: [metric = (mock score, cross-validated score, novel-taxa score)]. If two cut-offs are given, the second indicates a higher cut-off used to select parameters for the developmental NB-bespoke method, and the configurations listed are the union of the two cutoffs: the second cutoff for selecting NB-bespoke, the first for selecting all other methods

### Computational runtime

High-throughput sequencing platforms (and experiments) continue to yield increasing sequence counts, which—even after quality filtering and dereplication or operational taxonomic unit clustering steps common to most microbiome analysis pipelines—may exceed thousands of unique sequences that need classification. Increasing numbers of query sequences and references sequences may lead to unacceptable runtimes, and under some experimental conditions, the top-performing method (based on precision, recall, or some other metric) may be insufficient to handle large numbers of sequences within an acceptable time frame. For example, quick turnarounds may be vital under clinical scenarios as microbiome evaluation becomes translated to clinical practice, or commercial scenarios, when large sample volumes and client expectations may constrain turnaround times and method selection.

**Fig. 5:**
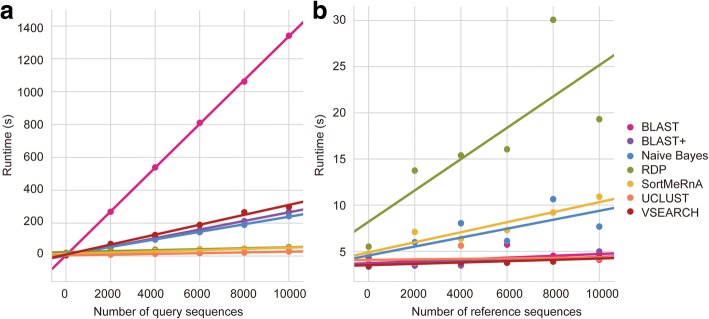
Runtime performance comparison of taxonomy classifiers. Runtime (s) for each taxonomy classifier either varying the number of query sequences and keeping a constant 10,000 reference sequences (**a**) or varying the number of reference sequences and keeping a constant 1 query sequence (**b**)

We assessed computational runtime as a linear function of (1) the number of query sequences and (2) the number of reference sequences. Linear dependence is empirically evident in Fig. 5. For both of these metrics, the slope is the most important measure of performance. The intercept may include the amount of time taken to train the classifier, preprocess the reference sequences, load preprocessed data, or other “setup” steps that will diminish in significance as sequence counts grow, and hence is negligible.

UCLUST (0.000028 s/sequence), VSEARCH (0.000072 s/sequence), BLAST+ (0.000080 s/sequence), and legacy BLAST (0.000100 s/sequence) all exhibit shallow slopes with increasing numbers of reference sequences. Naive Bayes (0.000483 s/sequence) and SortMeRNA (0.000543 s/sequence) yield moderately higher slopes and RDP (0.001696 s/sequence) demonstrates the steepest slope (Fig. 5b). For runtime as a function of query sequence count, UCLUST (0.002248 s/sequence), RDP (0.002920 s/sequence), and SortMeRNA (0.003819 s/sequence) have relatively shallow slopes (Fig. 5a). Naive Bayes (0.022984 s/sequence), BLAST+ (0.026222 s/sequence), and VSEARCH (0.030190 s/sequence) exhibit greater slopes. Legacy BLAST (0.133292 s/sequence) yielded a slope magnitudes higher than other methods, rendering this method impractical for large data sets.

## Discussion

We have developed and validated several machine-learning and alignment-based classifiers provided in q2-feature-classifier and benchmarked these classifiers, as well as other common classification methods, to evaluate their strengths and weaknesses for marker-gene amplicon sequence classification across a range of parameter settings for each (Table 2).

Each classifier required some degree of optimization to define top-performing parameter configurations, with the sole exception of QIIME 1’s legacy BLAST wrapper, which was unaffected by its only user-defined parameter, e-value, over a range of 10^− 10^ to 1000. For all other methods, performance varied widely depending on parameter settings, and a single method could achieve among the worst performance with one configuration but among the best performance with another. Configurations greatly affected accuracy with mock community, cross-validated, and novel taxon evaluations, indicating that optimization is necessary under a variety of performance conditions, and optimization for one condition may not necessarily translate to another. Mock community and cross-validated evaluations exhibited similar results, but novel taxon evaluations selected different optimal configurations for most methods (Fig. 4), indicating that configurations optimized to one condition, e.g., high-recall classification of known sequences, may be less suited for other conditions, e.g., classification of novel sequences. Table 2 lists the top-performing configuration for each method for several standard performance conditions.

Optimal configurations also varied among different evaluation metrics. Precision and recall, in particular, exhibited some mutual opposition, such that methods increasing precision reduced recall. For this reason, F-measure, the harmonic mean of precision and recall, is a useful metric for choosing configurations that are well balanced for average performance. “Balanced” method configurations—which maximize F-measure scores for mock, cross-validated, and novel taxon evaluations (Table 2)—are best suited for a wide range of user conditions. The naive Bayes classifier with k-mer lengths of 6 or 7 and confidence = 0.7 (or confidence ≥ 0.9 if using bespoke class weights), RDP with confidence = 0.6–0.7, and UCLUST (minimum consensus = 0.51, minimum similarity = 0.9, max accepts = 3) perform best under these conditions (Table 2). Performance is dramatically improved using bespoke class weights for 16S rRNA sequences (Fig. 4a, b), though this approach is developmental and only applicable when the expected composition of samples is known in advance (a scenario that is becoming increasingly common with the increasing quantity of public microbiome data, and which could be aided by microbiome data sharing resources such as Qiita (http://qiita.microbio.me)). For ITS sequences, the naive Bayes classifier with k-mer lengths of 6 or 7 and confidence ≥ 0.9, or RDP with confidence = 0.7–0.9, perform best, and the effects of bespoke class weights are less pronounced (Fig. 4c, d).

However, some users may require high-precision classifiers when false-positives may be more damaging to the outcome, e.g., for detection of pathogens in a sample. Precision scores are maximized by naive Bayes and RDP classifiers with high confidence settings (Table 2). Optimizing for precision will significantly damage recall by yielding a high number of false negatives.

Other users may require high-recall classifiers when false-negatives and underclassification hinder interpretation, but false positives (mostly overclassification to a closely related species) are less damaging. For example, in environments with high numbers of unidentified species, a high-precision classifier may yield large numbers of unclassified sequences; in such cases, a second pass with a high-recall configuration (Table 2) may provide useful inference of what taxa are most similar to these unclassified sequences. When recall is optimized, precision tends to suffer slightly (leading to similar F-measure scores to “balanced” configurations) but novel taxon classification accuracy is minimized, as these configurations tend to overclassify (Table 2). Any user prioritizing recall ought to be aware of and acknowledge these risks, e.g., when sharing or publishing their results, and understand that many of the species-level classifications may be wrong, particularly if the samples are expected to contain many uncharacterized species. For 16S rRNA gene sequences, naive Bayes bespoke classifiers with k-mer lengths between 12 and 32 and confidence = 0.5 yield maximal recall scores, but RDP (confidence = 0.5) and naive Bayes (uniform class weights, confidence = 0.5, k-mer length = 11, 12, or 18) also perform well (Table 2). Fungal recall scores are maximized by the same configurations recommended for “balanced” classification, i.e., naive Bayes classifiers with k-mer lengths of 6 or 7 and confidence between 0.92 and 0.98 or RDP with confidence between 0.7 and 0.9 (Table 2).

Runtime requirements may also be the chief concern dictating method selection for some users. QIIME 1’s UCLUST wrapper provides the fastest runtime while still achieving reasonably good performance for most evaluations; naive Bayes, RDP, and BLAST+ also delivered reasonably low runtime requirements and outperform UCLUST on most other evaluation metrics.

This study did not compare methods for classification of shotgun metagenome sequencing data sets, which present a series of unique challenges that do not exist for marker-gene amplicon sequence data. These include much higher unique sequence counts (making runtime a greater priority) and different analysis and quality control protocols. Metagenome sequences also exhibit heterogenous coverage and length, unlike marker-gene amplicon sequences, which typically have uniform start sites and read lengths within a single sequencing run. A recent benchmark of metagenome taxonomic profiling methods describes similar results to our benchmark of marker-gene sequence classifiers: most profilers perform well from phylum to family level but performance degrades at genus and species levels; different methods display superior performance according to different performance metrics; and parameter configuration dramatically impacts performance [26]. In the current study, we focused on benchmarking and optimizing classifiers for marker-gene amplicon sequence data, in light of the distinct needs of metagenome and marker-gene sequence datasets. Further testing is needed to assess the performance of these methods for metagenome sequence classification. Additional studies are also warranted to compare the performance of metagenome sequence classifiers for classification of marker-gene amplicon sequences. The tax-credit evaluation framework could facilitate this process, and we plan to continue to develop q2-feature-classifier to integrate methods that demonstrate superior performance for amplicon sequence classification.

We acknowledge several limitations to this study. First, we compare the q2-feature-classifier methods to the classifiers that have been most commonly used for classification of 16S rRNA and ITS marker-gene amplicon sequences accessed through QIIME 1 (RDP, BLAST, uclust, SortMeRNA). This study therefore focuses on classification methods that are implemented either in QIIME 1 or QIIME 2. We note that in many cases, QIIME wraps other implementations of these methods, and our results therefore should generalize beyond QIIME. Other methods—including metagenome sequence classifiers—deserve comparison. The tax-credit framework will support ongoing methods optimizations and comparisons to our foundational analysis by the microbiome research community. Second, the simulated sequence reads currently used in tax-credit do not incorporate sequencing errors, which limits their application for inferring classification performance under biological conditions. We instead use mock communities to assay classification of noisy sequence data and simulated data to assess idealized performance (i.e., independent of sequence errors). Mock communities also test actual experimental conditions (encompassing PCR, sequencing, and other technical biases that can be difficult to model), instead of attempting to simulate sequence errors, and hence we argue that the use of multiple testing datasets (mock, simulated cross-validated, and novel taxa simulations) is a strength of our study that allows us to query different aspects of classifier performance in isolation. However, this caveat—that our sequence simulations do not contain simulated errors—must be accounted for when interpreting those results.

## Conclusions

The classification methods provided in q2-feature-classifier will support improved taxonomy classification of marker-gene amplicon sequences, and are released as a free, open-source plugin for use with QIIME 2. We demonstrate that these methods perform as well as or better than other leading taxonomy classification methods on a number of performance metrics. The naive Bayes, VSEARCH, and BLAST+ consensus classifiers described here are released for the first time in QIIME 2, with optimized “balanced” configurations (Table 2) set as defaults.

We also present the results of a benchmark of several widely used taxonomy classifiers for marker-gene amplicon sequences and recommend the top-performing methods and configurations for the most common user scenarios. Our recommendations for “balanced” methods (Table 2) will be appropriate for most users who are classifying 16S rRNA gene or fungal ITS sequences, but other users may prioritize high-precision (low false-positive) or high-recall (low false-negative) methods.

We have also shown that great potential exists for improving the accuracy of taxonomy classifications by appropriately setting class weights for the machine learning classifiers. Currently, no tools exist that allow users to generate appropriate values for these class weights in real applications. Compiling appropriate class weights for different sample types could be a promising approach to further improve taxonomic classification of marker gene sequence reads.

## Methods

### Mock communities

All mock communities were sourced from mockrobiota [14]. Raw fastq files were demultiplexed and processed using tools available in QIIME 2 (version 2017.4) (https://qiime2.org). Reads were demultiplexed with q2-demux (https://github.com/qiime2/q2-demux) and quality filtered and dereplicated with q2-dada2 [4]. Representative sequence sets for each dada2 sequence variant were used for taxonomy classification with each classification method.

The inclusion of multiple mock community samples is important to avoid overfitting; optimizing method performance to a small set of data could result in overfitting to the specific community compositions or conditions under which those data were generated, which reduces the robustness of the classifier.

### Cross-validated simulated reads

The simulated reads used here were derived from the reference databases using the “Cross-validated classification performance” notebooks in our project repository. The reference databases were either Greengenes or UNITE (99% OTUs) that were cleaned according to taxonomic label to remove sequences with ambiguous or null labels. Reference sequences were trimmed to simulate amplification using standard PCR primers and slice out the first 250 bases downstream (3′) of the forward primer. The bacterial primers used were 27F/1492R [27] to simulate full-length 16S rRNA gene sequences, 515F/806R [28] to simulate 16S rRNA gene V4 domain sequences, and 27F/534R [29] to simulate 16S rRNA gene V1–3 domain sequences; the fungal primers used were BITSf/B58S3r [30] to simulate ITS1 internal transcribed spacer DNA sequences. The exact sequences were used for cross validation and were not altered to simulate any sequencing error; thus, our benchmarks simulate denoised sequence data [4] and isolate classifier performance from impacts from sequencing errors. Each database was stratified by taxonomy and 10-fold randomized cross-validation data sets were generated using scikit-learn’s library functions. Where a taxonomic label had less than 10 instances, taxonomies were amalgamated to make sufficiently large strata. If, as a result, a taxonomy in any test set was not present in the corresponding training set, the expected taxonomy label was truncated to the nearest common taxonomic rank observed in the training set (e.g., *Lactobacillus casei* would become *Lactobacillus*). The notebook detailing simulated read generation (for both cross-validated and novel taxon reads) prior to taxonomy classification is available at https://github.com/caporaso-lab/tax-credit-data/blob/0.1.0/ipynb/novel-taxa/dataset-generation.ipynb.

Classification performance was also slightly modified from a standard machine-learning scenario as the classifiers in this study are able to refuse classification if they are not confident above a taxonomic level for a given sample. This also accommodates the taxonomy truncation that we performed for this test. The methodology was consistent with that used below for novel taxon evaluations, so we defer its description to the next section.

### “Novel taxon” simulation analysis

“Novel taxon” classification analysis was performed to test the performance of classifiers when assigning taxonomy to sequences that are not represented in a reference database, e.g., as a simulation of what occurs when a method encounters an undocumented species [22–25]. In this analysis, simulated amplicons were filtered from those used for the cross-validation analysis. For all sequences present in each test set, sequences sharing taxonomic affiliation at a given taxonomic level L (e.g., to species level) in the corresponding training set were removed. Taxa are stratified among query and test sets such that for each query taxonomy at level L, no reference sequences match that taxonomy, but at least one reference sequence will match the taxonomic lineage at level L-1 (e.g., same genus but different species). An ideal classifier would assign taxonomy to the nearest common taxonomic lineage (e.g., genus), but would not “overclassify” [25] to near neighbors (e.g., assign species-level taxonomy when species X is removed from the reference database). For example, a “novel” sequence representing the species *Lactobacillus brevis* should be classified as “*Lactobacillus,*” without species-level annotation, in order to be considered a true positive in this analysis. As described above for cross-validated reads, these novel taxa simulated communities were also tested in both bacterial (B) and fungal (F) databases on simulated amplicons trimmed to simulate 250-nt sequencing reads.

Novel taxon classification performance is evaluated using precision, recall, F-measure, overclassification rates, underclassification rates, and misclassification rates [25] for each taxonomic level (phylum to species), computed with the following definitions (see below, *Performance analyses using simulated reads*, for full description of precision, recall, and F-measure calculations):

1. A true positive is considered the nearest correct lineage contained in the reference database. For example, if *Lactobacillus brevis* is removed from the reference database and used as a query sequence, the only correct taxonomy classification would be “*Lactobacillus,*” without species-level classification.
2. A false positive would be either a classification to a different *Lactobacillus* species (*overclassification*) or any genus other than *Lactobacillus* (*misclassification*).
3. A false negative occurs if an expected taxonomy classification (e.g., “*Lactobacillus*”) is not observed in the results. Note that this will be the modified taxonomy expected when using a naive reference database and is not the same as the true taxonomic affiliation of a query sequence in the novel taxa analysis. A false negative results from misclassification, overclassification, or when the classification contains the correct basal lineage, but does not assign a taxonomy label at level L (*Underclassification*), e.g., classification as “*Lactobacillaceae*,” but no genus level classification.

### Taxonomy classification

Representative sequences for all analyses (mock community, cross-validated, and novel taxa) were classified taxonomically using the following taxonomy classifiers and setting sweeps:

1. q2-feature-classifier multinomial naive Bayes classifier. Varied k-mer length in {4, 6, 7, 8, 9, 10, 11, 12, 14, 16, 18, 32} and confidence threshold in {0, 0.5, 0.7, 0.9, 0.92, 0.94, 0.96, 0.98, 1}.
2. BLAST+ [9] local sequence alignment followed by consensus taxonomy classification implemented in q2-feature-classifier. Varied max accepts from 1 to 100; percent identity from 0.80 to 0.99; and minimum consensus from 0.51 to 0.99. See description below.
3. VSEARCH [10] global sequence alignment followed by consensus taxonomy classification implemented in q2-feature-classifier. Varied max accepts from 1 to 100; percent identity from 0.80 to 0.99; and minimum consensus from 0.51 to 0.99. See description below.
4. Ribosomal Database Project (RDP) naïve Bayesian classifier [11] (QIIME1 wrapper), with confidence thresholds between 0.0 and 1.0 in steps of 0.1.
5. Legacy BLAST [15] (QIIME1 wrapper) varying e-value thresholds from 1e-9 to 1000.
6. SortMeRNA [13] (QIIME1 wrapper) varying minimum consensus fraction from 0.51 to 0.99; similarity from 0.8 to 0.9; max accepts from 1 to 10; and coverage from 0.8 to 0.9.
7. UCLUST [12] (QIIME1 wrapper) varying minimum consensus fraction from 0.51 to 0.99; similarity from 0.8 to 0.9; and max accepts from 1 to 10.

With the exception of the UCLUST classifier, we have only benchmarked the performance of open-source, free, marker-gene-agnostic classifiers, i.e., those that can be trained/aligned on a reference database of *any* marker gene. Hence, we excluded classifiers that can only assign taxonomy to a particular marker gene (e.g., only bacterial 16S rRNA genes) and those that rely on specialized or unavailable reference databases and cannot be trained on other databases, effectively restricting their use for other marker genes and custom databases.

Classification of bacterial/archaeal 16S rRNA gene sequences was made using the Greengenes (13_8 release) [5] reference sequence database preclustered at 99% ID, with amplicons for the domain of interest extracted using primers 27F/1492R [27], 515F/806R [28], or 27F/534R [29] with q2-feature-classifier’s extract_reads method. Classification of fungal ITS sequences was made using the UNITE database (version 7.1 QIIME developer release) [31] preclustered at 99% ID. For the cross validation and novel taxon classification tests, we prefiltered to remove sequences with incomplete or ambiguous taxonomies (containing the substrings ‘unknown,’ ‘unidentified,’ or ‘_sp’ or terminating at any level with ‘__’).

The notebooks detailing taxonomy classification sweeps of mock communities are available at https://github.com/caporaso-lab/tax-credit-data/tree/0.1.0/ipynb/mock-community. Cross-validated read classification sweeps are available at https://github.com/caporaso-lab/tax-credit-data/blob/0.1.0/ipynb/cross-validated/taxonomy-assignment.ipynb. Novel taxon classification sweeps are available at https://github.com/caporaso-lab/tax-credit-data/blob/0.1.0/ipynb/novel-taxa/taxonomy-assignment.ipynb.

### Runtime analyses

The tax-credit framework employs two different runtime metrics: as a function of (1) the number of query sequences or (2) the number of reference sequences. Taxonomy classifier runtimes were logged while performing classifications of pseudorandom subsets of 1, 2000, 4000, 6000, 8000, and 10,000 sequences from the Greengenes 99% OTU database. Each subset was drawn once then used for all of the tests as appropriate. All runtimes were computed on the same Linux workstation (Ubuntu 16.04.2 LTS, Intel Xeon CPU E7–4850 v3 @ 2.20GHz, 1TB memory). The exact commands used for runtime analysis are presented in the “Runtime analyses” notebook in the project repository (https://github.com/caporaso-lab/tax-credit-data/blob/0.1.0/ipynb/runtime/analysis.ipynb).

### Performance analyses using simulated reads

Cross-validated and novel taxa reads are evaluated using the classic precision, recall, and F-measure metrics [5] (novel taxa use the standard calculations as described below, but modified definitions for true positive (*TP*), false positive (*FP*), and false negative (*FN*), as described above for novel taxon classification analysis).

Precision, recall, and F-measure are calculated as follows:

- *Precision* = *TP/(TP* + *FP)* or the fraction of sequences that were classified correctly at level L.
- *Recall* = *TP/(TP* + *FN)* or the fraction of expected taxonomic labels that were predicted at level L.
- *F-measure* = 2 × precision × recall/(precision+recall), or the harmonic mean of precision and recall.

The Jupyter notebook detailing commands used for evaluation of cross-validated read classifications is available at https://github.com/caporaso-lab/tax-credit-data/blob/0.1.0/ipynb/cross-validated/evaluate-classification.ipynb. The notebook for evaluation of novel taxon classifications is available at https://github.com/caporaso-lab/tax-credit-data/blob/0.1.0/ipynb/novel-taxa/evaluate-classification.ipynb.

### Performance analyses using mock communities

The Jupyter notebook detailing commands used for evaluation of mock communities, including the three evaluation types described below, is available at https://github.com/caporaso-lab/tax-credit-data/blob/0.1.0/ipynb/mock-community/evaluate-classification-accuracy.ipynb.

### Precision and recall

Classic precision, recall, and F-measure are used to calculate mock community classification accuracy, using the definitions given above for simulated reads. These metrics require knowing the expected classification of each sequence, which we determine by performing a gapless alignment between each representative sequence in the mock community and the marker-gene sequences of each microbial strain added to the mock community. These “expected sequences” are provided for the mock communities in mockrobiota [14]. Representative sequences are assigned the taxonomy of the best alignment, and any representative sequence with more than three mismatches to the expected sequences are excluded from precision/recall calculations. If a representative sequence aligns to more than one expected sequence equally well, all top hits are accepted as the “correct” classification. This scenario is rare and typically only occurred when different strains of the same species were added to the same mock community to intentionally produce this challenge (e.g., for mock-12 as described by [4]). Precision, recall, and F-measure are then calculated by comparing the “expected” classification for each mock community sequence to the classifications predicted by each taxonomy classifier using the full reference databases, as described above.

### Taxon accuracy rate and taxon detection rate

Taxon accuracy rate (TAR) and taxon detection rate (TDR) are used for qualitative compositional analyses of mock communities. As the true taxonomy labels for each sequence in a mock community are not known with absolute certainty, TAR and TDR are useful alternatives to precision and recall that instead rely on the presence/absence of expected taxa, or microbiota that are intentionally added to the mock community. In practice, TAR/TDR are complementary metrics to precision/recall and should provide similar results if the expected classifications for mock community representative sequences are accurate.

At a given taxonomic level, a classification is a

- True positive (*TP*), if that taxon is both observed and expected.
- False positive (*FP*), if that taxon is observed but not expected.
- False negative (*FN*), if a taxon is expected but not observed.

These are used to calculate TAR and TDR as

- *TAR* = *TP/(TP* + *FP)* or the fraction of observed taxa that were expected at level L.
- *TDR* = *TP/(TP* + *FN)* or the fraction of expected taxa that are observed at level L.

### Bray-Curtis dissimilarity

Bray-Curtis dissimilarity [32] is used to measure the degree of dissimilarity between two samples as a function of the abundance of each species label present in each sample, treating each species as equally related. This is a useful metric for evaluating classifier performance by assessing the relative distance between each predicted mock community composition (abundance of taxa in a sample based on results of a single classifier) and the expected composition of that sample. For each classifier, Bray-Curtis distances between the expected and observed taxonomic compositions are calculated for each sample in each mock community dataset; this yields a single expected-observed distance for each individual observation. The distance distributions for each method are then compared statistically using paired or unpaired t-tests to assess whether one method (or configuration) performs consistently better than another.

### New taxonomy classifiers

We describe q2-feature-classifier (https://github.com/qiime2/q2-feature-classifier), a plugin for QIIME 2 (https://qiime2.org/) that performs multi-class taxonomy classification of marker-gene sequence reads. In this work, we compare the consensus BLAST+ and VSEARCH methods and the naive Bayes scikit-learn classifier. The software is free and open-source.

### Machine learning taxonomy classifiers

The q2-feature-classifier plugin allows users to apply any of the suite of machine learning classifiers available in scikit-learn (http://scikit-learn.org) to the problem of taxonomy classification of marker-gene sequences. It functions as a lightweight wrapper that transforms the problem into a standard document classification problem. Advanced users can input any appropriate scikit-learn classifier pipeline, which can include a range of feature extraction and transformation steps as well as specifying a machine learning algorithm.

The plugin provides a default method which is to extract k-mer counts from reference sequences and train the scikit-learn multinomial naive Bayes classifier, and it is this method that we test extensively here. Specifically, the pipeline consists of a sklearn.feature_extraction.text.HashingVectorizer feature extraction step followed by a sklearn.naive_bayes.MultinomialNB classification step. The use of a hashing feature extractor allows the use of significantly longer k-mers than the 8-mers that are used by RDP Classifier, and we tested up to 32-mers. Like most scikit-learn classifiers, we are able to set class weights when training the multinomial naive Bayes classifiers. In the naive Bayes setting, setting class weights means that class priors are not derived from the training data or set to be uniform, as they are for the RDP Classifier. For more details on how class weights enter the calculations, please refer to the scikit-learn User Guide (http://scikit-learn.org).

In most settings, it is highly unlikely that the assumption of uniform weights is correct. That assumption is that each of the taxa in the reference database is equally likely to appear in each sample. Setting class weights to more realistic values can greatly aid the classifier in making more accurate predictions, as we show in this work. When testing the mock communities, we made use of the fact that the sequence compositions were known a priori for the bespoke classifier. For the simulated reads studies, we allowed the classifier to set the class weights from the class frequencies observed in each training set for the bespoke classifier.

For this study, we performed two parameter sweeps on the mock communities: an initial broad sweep to optimize feature extraction parameters and then a more focused sweep to optimize k-mer length and confidence parameter settings. These sweeps included varying the assumptions regarding class weights. The focused sweeps were also performed for the cross-validated and novel taxa evaluations, but only for the assumption of uniform class priors. The results for the focused sweeps across all data sets are those which are compared against the other classifiers in this work.

The broad sweeps used a modified scikit-learn pipeline which consisted of the sklearn.feature_extraction.text.HashingVectorizer followed by the sklearn.feature_extraction.text.TfidfTransformer, then the sklearn.naive_bayes.MultinomialNB. We performed a full grid search over the parameters shown in Table 3. The conclusion from the initial sweep was that the TfidfTransformer step did not significantly improve classification that n_features should be set to 8192, that feature vectors should be normalized using L2 normalization, and that the alpha parameter for the naive Bayes classifier should be set to 0.001. Please see https://github.com/caporaso-lab/tax-credit-data/blob/0.1.0/ipynb/mock-community/evaluate-classification-accuracy-nb-extra.ipynb for details.

**Table 3:** Naive Bayes broad grid search parameters

Step	Parameter	Values
sklearn.feature_extraction.text.HashingVectorizer	n_features	1024, 8192, 65,536
	ngram_range	[4,4], [8, 8], [16, 16], [4,16]
sklearn.feature_extraction.text.TfidfTransformer	norm	l1, l2, None
	usd_idf	True, False
sklearn.naive_bayes.MultinomialNB	alpha	0.001, 0.01, 0.1
	class_prior	None, array of class weights
post processing	confidence	0, 0.2, 0.4, 0.6, 0.8

### Consensus taxonomy alignment-based classifiers

Two new classifiers implemented in q2-feature-classifier perform consensus taxonomy classification based on alignment of a query sequence to a reference sequence. The methods classify_consensus_vsearch and classify_consensus_blast use the global aligner VSEARCH [10] or the local aligner BLAST+ [9], respectively, to return up to maxaccepts reference sequences that align to the query with at least perc_identity similarity. A consensus taxonomy is then assigned to the query sequence by determining the taxonomic lineage on which at least min_consensus of the aligned sequences agree. This consensus taxonomy is truncated at the taxonomic level at which less than min_consensus of taxonomies agree. For example, if a query sequence is classified with maxaccepts = 3, min_consensus = 0.51, and the following top hits:

k__Bacteria; p__Firmicutes; c__Bacilli; o__Lactobacillales; f__Lactobacillaceae; g__Lactobacillus; s__brevis.

k__Bacteria; p__Firmicutes; c__Bacilli; o__Lactobacillales; f__Lactobacillaceae; g__Lactobacillus; s__brevis.

k__Bacteria; p__Firmicutes; c__Bacilli; o__Lactobacillales; f__Lactobacillaceae; g__Lactobacillus; s__delbrueckii.

The taxonomy label assigned will be k__Bacteria; p__Firmicutes; c__Bacilli; o__Lactobacillales; f__Lactobacillaceae; g__Lactobacillus; s__brevis. However, if min_consensus = 0.99, the taxonomy label assigned will be k__Bacteria, p__Firmicutes, c__Bacilli, o__Lactobacillales, f__Lactobacillaceae, and g__Lactobacillus.
